# Reconfigurable MIMO-based self-powered battery-less light communication system

**DOI:** 10.1038/s41377-024-01566-3

**Published:** 2024-08-28

**Authors:** Jose Ilton De Oliveira Filho, Abderrahmen Trichili, Omar Alkhazragi, Mohamed-Slim Alouini, Boon S. Ooi, Khaled Nabil Salama

**Affiliations:** 1 Computer, Electrical and Mathematical Sciences & Engineering, King Abdullah University of Science and Technology, Thuwal, Saudi Arabia; 2 NEOM Institute for Ocean Science and Solutions, Neom, Thuwal, Saudi Arabia

**Keywords:** Optoelectronic devices and components, Fibre optics and optical communications

## Abstract

Simultaneous lightwave information and power transfer (SLIPT), co-existing with optical wireless communication, holds an enormous potential to provide continuous charging to remote Internet of Things (IoT) devices while ensuring connectivity. Combining SLIPT with an omnidirectional receiver, we can leverage a higher power budget while maintaining a stable connection, a major challenge for optical wireless communication systems. Here, we design a multiplexed SLIPT-based system comprising an array of photodetectors (PDs) arranged in a 3 × 3 configuration. The system enables decoding information from multiple light beams while simultaneously harvesting energy. The PDs can swiftly switch between photoconductive and photovoltaic modes to maximize information transfer rates and provide on-demand energy harvesting. Additionally, we investigated the ability to decode information and harvest energy with a particular quadrant set of PDs from the array, allowing beam tracking and spatial diversity. The design was explored in a smaller version for higher data rates and a bigger one for higher power harvesting. We report a self-powering device that can achieve a gross data rate of 25.7 Mbps from a single-input single-output (SISO) and an 85.2 Mbps net data rate in a multiple-input multiple-output (MIMO) configuration. Under a standard AMT1.5 illumination, the device can harvest up to 87.33 mW, around twice the power needed to maintain the entire system. Our work paves the way for deploying autonomous IoT devices in harsh environments and their potential use in space applications.

## Abstract (web summary)

Combining space and time-domain SLIPT enables a self-powered light communication system reaching up to 85.2 Mbps, with potential applications in underwater and space sectors.

## Graphical abstract

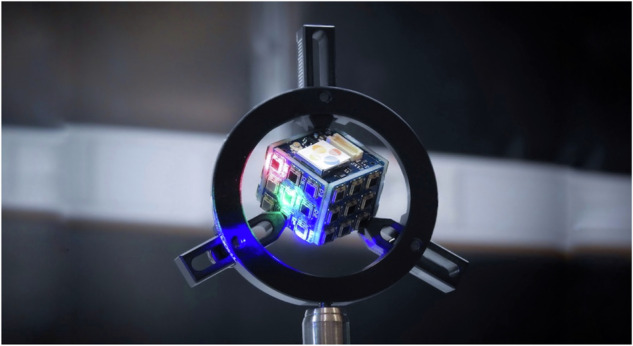

## Introduction

There has been considerable attention on the simultaneous wireless information and power transfer (SWIPT)^1,2^. SWIPT harnesses ambient radio frequency (RF) waves, commonly used for communication, to power or extend the battery life of low-power IoT devices, eliminating the need for frequent battery replacements and addressing a critical bottleneck in the widespread adoption of IoT. Compared to many reported wireless power transfer (WPT) demonstrations^3–5^, a major advantage of SWIPT is its ability to utilize existing RF infrastructure^6–8^. However, SWIPT faces the challenges of low energy conversion efficiency and complicated receiver design^7^. In contrast, simultaneous lightwave information and power transfer (SLIPT), serving as the optical counterpart of SWIPT, is an emerging technology that benefits from the broad unlicensed optical spectrum and the utilization of low-cost, and off-the-shelf components. SLIPT, as a *piggybacked* feature on optical wireless communication, makes it highly valuable for various applications, including indoor^9^, outdoor^10–12^, and underwater^13^ scenarios. Using solar cells (SCs) of different materials to receive information and harvest energy is the key feature for all previous demonstrations. A seminal experimental demonstration reported decoding visible light signals at a low data rate of 3 kbps while harvesting solar energy using the same SC^14^. An SC-based receiver was designed to establish a short communication link of tens of cm with a data rate of 11.84 Mbps while generating two mW of power^15^. An organic SC with an active area of 8 mm^2^ was used to harvest 0.43 W of power and achieve a data rate of 34.2 Mbps^16^. A data rate of 522 Mbps was demonstrated over a 2-m link using a GaAs SC with an active area less than 1-mm^2^ and illuminated with light signals at 847 nm^17^. Using a perovskite SC with a 6.5 mm^2^ active area, a 660-nm LD-based 40-cm long link was demonstrated, reporting an average data rate of 49 Mbps and highlighting the ability to harvest between 3 and 5 mW of power^18^.

Most of these demonstrations rely on a time-splitting approach consisting of using the same receiver to harvest energy and decode information over different time frames. In addition to the time-splitting mechanism, there are two other main strategies for SLIPT: power splitting and space splitting. Power splitting involves dividing the power of an incoming light beam, allocating a portion for information decoding and the remaining portion for energy harvesting. Space splitting utilizes spatially separated receivers for energy harvesting and information decoding operations. Space splitting can potentially allow for the creation of omnidirectional optical wireless communication (OWC) receivers that have long been a topic of interest^19^. Using spatially separated receivers also enables angle-of-arrival and position estimation^20^. Light beam tracking can mitigate other common OWC problems, such as transmitter and receiver misalignment^21^.

Several studies have addressed easing the precise alignment constraints of OWC links, notably through the use of mechanical beam steering^22,23^, arrays of receivers^24,25^, luminescent solar concentrators that are planar^26^ or in the form of scintillating fibers^27,28^, and receivers with angle diversity^29,30^. Space splitting can relax the alignment by simply creating multiple receivers pointing in different directions.

**Fig. 1:**
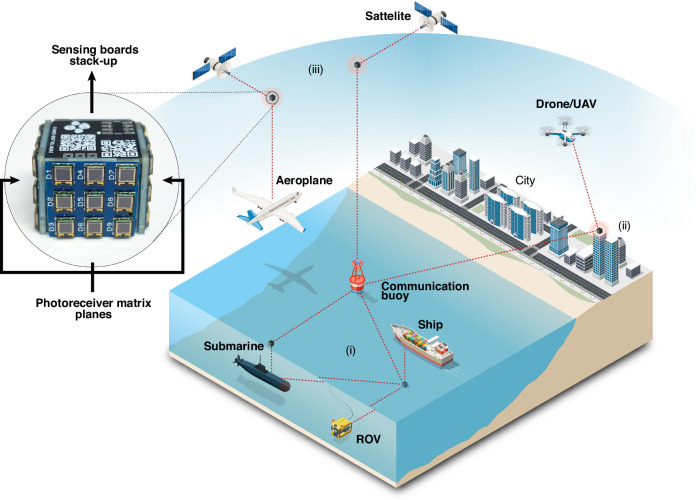
**Conceptual illustration of the SLIPT-based cube-shaped omnidirectional receiver**. Cube-shaped receiver used in (i) shallow water environment, (ii) open-air, and (iii) in space

Furthermore, spatially separated receivers can employ diversity schemes, which are widely used in RF communication through multiple input/multiple output (MIMO) systems. Using MIMO for spatial diversity in optical communication systems has been theoretically studied^31^. Yet the full potential of OWC MIMO configurations that can be used across a wide range of applications still needs to be demonstrated. The design of a device that addresses various constraints of OWC links and could be employed in different scenarios is illustrated in Fig. 1.

In this work, to the best of our knowledge, we propose the first system that combines space and time-domain SLIPT, creating an energy-harvesting MIMO-SLIPT system. The designed system can be programmed to operate depending on the received wavelength through a concept we refer to as the wavelength-based SLIPT approach. Our proof-of-concept device can self-power, eliminating the need for an external power source. The developed system comprises a 3 × 3 silicon PD matrix, in which the PDs can be controlled independently to decode information or harvest energy. As a feature, the proposed device provides a mitigation solution for beam wandering, jittering, and misalignment issues for OWC links. We report communication tests using an orthogonal frequency-division multiplexing (OFDM) modulation scheme with net data rates exceeding 85 megabits per second (Mbps). At the same time, the device harvests enough energy for self-powering while supporting the operation of additional sensors. The various tested capabilities of the system are presented, and potential improvements are discussed.

Moreover, we name a single plane a two-dimensional (2D) space receiver and a multi-plane a three-dimensional (3D) space receiver. Both systems have multiple inputs and are capable of delivering multiple outputs. This MIMO concept allows for a high-speed connection or interrupted data stream from various sources. Our approach also introduces the concept of quadrant decoding for the SLIPT system. A total of 10 quadrant configurations can be set for each plane, thus making the device a reconfigurable 2D receiver analog to a programmable antenna array for diversity schemes.

## Results

### Multi-plane multiplexed system design

Easing the alignment and pointing requirements for OWC-SLIPT is possible using large-area solar panels^13^. However, this approach limits the receiver bandwidth, as solar panels are not usually configured in photo-conductive mode to maximize DC performance. The photo-conductive mode is essential to obtain higher frequency, as this mode is based on widening the depletion region by reverse biasing the receiver, thus making it more sensitive with a higher 3-dB bandwidth due to the lower junction capacitance and the shorter transient time resulting from the higher drift velocity. Nevertheless, a solution for this problem was introduced by simply mechanically switching the receiver connection, alternating between photo-conductive and photovoltaic mode depending on the application^10,13^. Although proven to be an efficient solution for SLIPT application, mechanical systems are prone to failure due to fatigue and are slower than electronic-based systems, despite notable advances in the field^32^. The electronic switch matrix also offers easy miniaturization, creating the opportunity to integrate multiple receivers with independent control. In this work, we leverage this advantage by integrating multiple small PDs with higher bandwidth than solar panels. The configuration creates a large receiver plane similar to a solar panel that can switch each PD unit to photo-conductive and photovoltaic modes. By having multiple independent interfaced PDs, the unit receivers can be grouped in quadrants to fulfill a diversity scheme. This concept allows for decoding independent data signals from various sources or collecting duplicated signals for maximal ratio combining (MRC). The quadrant concept has been demonstrated for indoor visible light positioning^20^. Using an array of PDs can enable MIMO communication similar to what has been presented in a previous study through organic dual-function photovoltaic cells^11^. For instance, each PD can decode the data from one particular source, increasing the overall data rate.

**Fig. 2:**
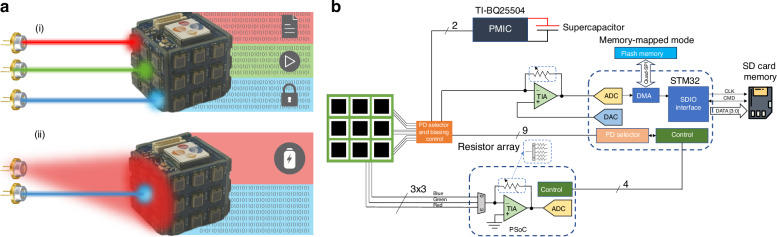
**A 3D SLIPT system with multiplexed receiver planes**. **a** The system receives simultaneously multiple data inputs throughout (i) different wavelengths and (ii) a single stream with a secondary beam for powering. **b** Circuit diagram of the system

As shown in Fig. 2a, one or multiple modulated laser beams can transmit power and data simultaneously. As the beams can be from different wavelengths, three wavelength detectors were added, each with three color channels. The color detection was designed to be done automatically at the hardware level, using an integrated analog and digital circuitry designed using Programmable System-on-Chip (PSoC). In this way, there is no processing burden, and the main microcontrollers (MCU) can use the power to process and decode the information with a bigger power budget. Although PSoC has internal components that can interface the PDs, its 3 dB bandwidth is limited to around 1 MHz, much lower than the large and small PDs used. For that, we utilized an external TIA connected directly to the output of the PD selector and biasing control. As the maximum data rate of our system is higher than what the MCU can process for decoding, a large external memory was added and interfaced using the SDIO interface, providing a maximum capable bandwidth of 100 Mbps. The simplified circuit diagram is presented in Fig. 2b.

The designed system presents two operational modes, single PD and quadrant PD mode. In the single PD mode, the emphasis is on maximizing the amount of harvested power. In this mode, eight PDs from the device continuously harvest energy, while only one PD is used to receive information signals. This mode ensures that a significant amount of power is harvested while maintaining a communication link. The primary advantage of the single PD mode is its higher power harvesting capability. Meanwhile, the quadrant mode prioritizes data transfer efficiency, in which up to four PDs can be assigned to receive information, thereby increasing the data throughput and optical link stability. However, the number of PDs used for energy harvesting is reduced to five. This trade-off results in a higher data transfer rate at the expense of slightly lower power harvesting capabilities. Another disadvantage of the quadrant mode is the higher resources needed for decoding, as the quadrant mode employs a maximum of four transimpedance amplifiers (TIA) in parallel, requiring a faster ADC or multiple ADCs in parallel.

### Single PD communication performance

**Fig. 3:**
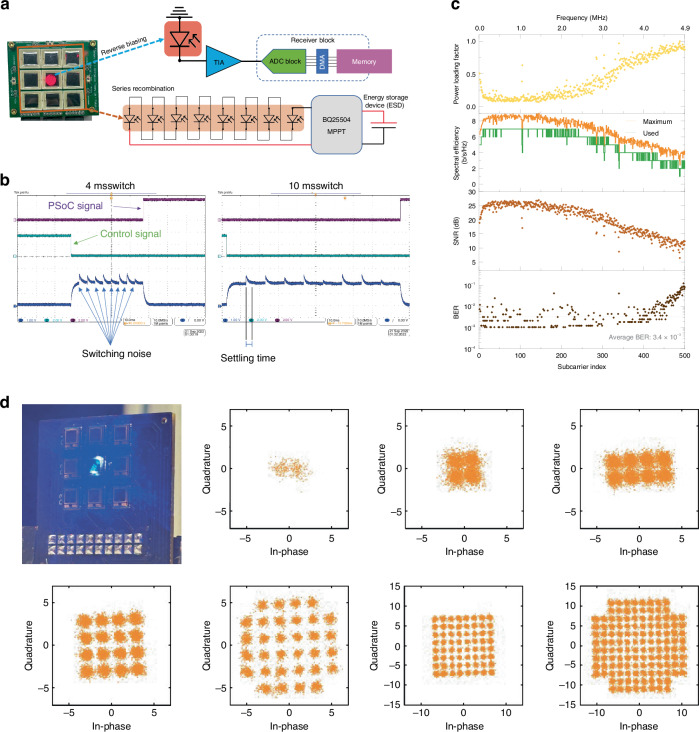
**Single PD configuration**. **a** Picture of the large receiver plane with a 658 nm laser beam and its system diagram for the single PD reverse biasing to maximum bandwidth achievement and recombination of all other PDs. **b** Oscillogram of MCU interruption command for best SNR scan throughout all PDs with 4 and 5 ms switching time. **c** Power loading factor, spectral efficiency, SNR, and BER per subcarrier. **d** A picture of the small receiver plane with a 450 nm laser beam modulated using quadrature amplitude modulation and its experimental results shown in the constellation

When the system, illustrated in Fig. 3a, is set to receive communication signals, the STM32 sends a control signal to the PD selector and biasing control block to reverse bias all PDs, one at a time. Following that, the STM32 utilizes its analog-to-digital converter (ADC) to sample the received signal from each PD. By comparing the signal-to-noise ratio (SNR) obtained from each PD, the STM32 identifies the PD that provides the highest SNR. Due to noise injected from the analog switch during the reverse bias operation, a settling time wait is implemented to mitigate interference with the ADC measurement. The total time for a full SNR scan takes up to 43 ms. This duration includes the settling time for each PD and the time required for the STM32 to perform the necessary calculations and comparisons. Figure 3b shows the control signal, the switching output signal, and the PSoC-ready signal. The switching between PDs in a multi-input single-receiver operation takes up to 22 *µs*. This maximum speed is derived from experimental measurement (see Fig. S1 and S2), which takes into account the recovery of the signal. Using an OFDM modulation scheme with bit and power loading to leverage the channel optimally, we obtained a gross data rate of 5.3 Mbps and a net data rate of 4 Mbps for the large area PD and a gross data rate of 25.7 Mbps with a net data rate of 21.3 Mbps for the small PD. Depending on the available SNR at each subcarrier, the number of bits per symbol can be adapted to achieve the highest possible throughput through a bit-loading process, and the power of each subcarrier is optimized depending on the SNR profile. For the large PD, the average measured bit error rate (BER) is 3.3 × 10^−3^, which is below the forward error correction (FEC) limit of 3.8 × 10^−3^. For the small PD, the average measured bit error rate (BER) is 3.4 × 10^−3^. Power loading factor, spectral efficiency, SNR, and BER per subcarrier are shown in Fig. 3(c). The constellation signal result is presented in Fig. 3d, in which the maximum QAM order used in our demonstration is 64. It is also noted a small SNR in the lower frequencies of the OFDM-QAM used; this is due to a low-pass filter used in the system to block the DC signal, mitigating the saturation of the TIA and ADC in the receiver circuitry. As a self-powered device, the saturation of its components for communication is a major problem if left unchecked.

### Quadrant-based decoding

**Fig. 4:**
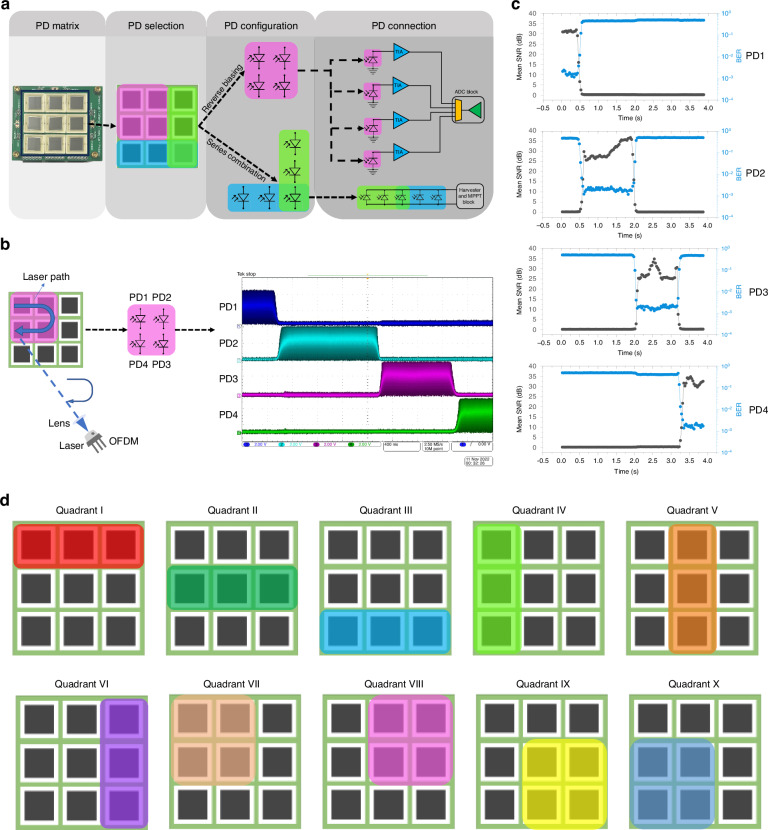
**Quadrant decoding configuration and results**. **a** Picture of the large receiver plane and its steps for PD quadrant recombination with four PDs going into reverse biasing and five PDs into series configuration. **b** Laser path and PDs output due to the laser path starting from PD1 and finishing in PD4. **c** SNR and BER of the OFDM signal versus time from each PD measured simultaneously. **d** The quadrants that can be configured using the system with a maximum simultaneous configuration of 4 PDs

Quadrant decoding refers to the method of receiving parallel data through multiple beams or a single one, either from a wandering beam or a beam spreading, for overall signal quality. For the quadrant configuration, a total of four steps are needed, Fig. 4a. In the first and second steps, an idle matrix is divided into quadrants for energy harvesting and information decoding. Afterward, all the PDs selected for energy harvesting are combined in series using the switching matrix, while the chosen PDs for information decoding are isolated and connected in parallel. These four parallel channels can reach a maximum of 85.2 Mbps of combined net data rate. In this configuration, the amount of energy harvested by the PDs can not power the entire system, as the MCU needs to run at full clock speed with four parallel signal streams being decoded. In this setup, where the PDs application is locked to quadrants, there’s only one switching reconfiguration needed, and it happens at the same top speed of 22 *µs*. This is the same speed as the single PD configuration because all the switches receive the command simultaneously. For demonstration, we set quadrant II for data while the laser drifted from PD1 to PD4. The laser continuously transmitted information throughout the drifting using an OFDM QAM-16 modulated signal. Figure 4b shows the signal received from PD1-4 in parallel while the laser’s signal was captured once it passed in front of each PD. Figure 4c shows the final processed SNR and BER for each PD. The SNR raises while the laser beam gets aligned with the PD, reaching its maximum value, and then the laser drifts away from the PD, leading to a gradual decrease of SNR. For long-distance applications, the divergence angle of the beams will significantly impact the MIMO schemes for a single-plane receiver. A typical visible laser with a large divergence angle will lead to an overlapping of the beams on the quadrant PDs. However, this effect is not perceived if the multi-plane receiver is used. We further investigated the impacts of beam incidence angle on communication efficiency and tracking, and the results and discussion are presented in Fig. S12 in the SI section Self-powering and tracking under different illumination angles.

### Wavelength switching

**Fig. 5:**
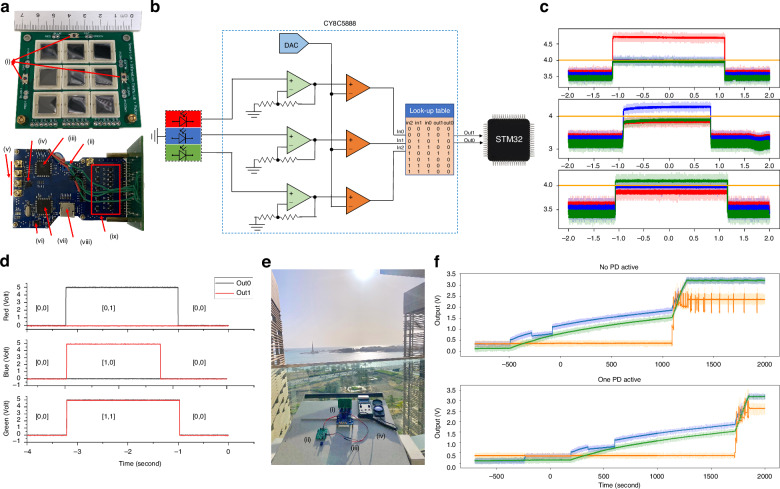
**Wavelength detection and energy harvesting results**. **a** Picture of the large area PD receiver panel with the (i) three-color detector and the full controller board showing the (ii) harvesting energy output, (iii) PSoC, (iv) TIAs, (v) four external TIA outputs, (vi) flash memory, (vii) STM32 MCU, (viii) external SD connector, and (ix) switching matrix. **b** Block diagram of the color detection circuit with hardware design implemented in the PSoC CY8C5888. **c** Output voltages from the PGA of each color detector under the illumination of a red laser, blue laser, and green LED. **d** Two-bit digital output signal. **e** Outdoor testing setup with the (i) self-powered device facing direct sunlight with its (ii) energy harvesting circuit (iii) in closed-loop sourcing the device, (iv) light meter. **f** Voltage of receiver panel output (orange), load/system (blue), and energy storage device (green) under single PD and No active PD configuration

The wavelength detection capability was tested as each laser beam frequency can be tuned for distinct functions that are optimum for each wavelength. Three wavelengths were used in the test: 450 nm (blue), 658 nm (red), and 530 nm (green). The PD used has a typical responsivity of 0.36 A/W at 630 nm, which decreases to nearly zero A/W with the wavelength decreases. This characteristic allows the red wavelengths (625–750 nm) to be used for power transfer. The green wavelengths (500–565 nm) exhibit higher responsivity than blue wavelengths (450–485 nm), making them more suitable for visible-light communication with the presented system. However, in underwater communication scenarios, blue wavelengths are more suitable due to lower absorption in water. Figure 5b shows the circuit diagram of a single color sensor system implemented in hardware. The design takes three clock cycles to provide the information of which color is detected through the two-bit output interface. At a 48 MHz clock, the design consumes 69.36 mW, and at a 3 MHz clock, the design consumes 27.88 mW. By sampling for 1 ms and sleeping for 200 ms, the color detection system consumes 139.4 *µJ*. Figure 5c, d show the output voltage of the PGA and the digital output, respectively. The threshold voltage generated by the internal DAC determines when the comparator output is high. The LUT outputs ‘[0,1]’ when the red wavelength is higher, ‘[1,0]’ when the blue wavelength is higher, ‘[1,1]’ when the green wavelength is higher, and ‘[0,0]’ for any other case.

### Energy harvesting and battery-less operation

In parallel decoding for the small PD plane, each PD channel can receive a much faster data rate than the MCU can process. A pass-through system architecture can be used to handle the data directly from the DMA to the external memory; however, even at maximum clock speed, a combined parallel data rate of 85.2 Mbps is not feasible using an ultra-low-power MCU. Nevertheless, using such a low-power processing unit enables the handling of the slower PD plane while being completely self-powered by the receiver plane without needing a battery. The targeted application will dictate the trade-off between processing power and battery-less features. There is a fundamental difference in the amount of power that can be harvested from sunlight and artificial light sources. For free-space optical communication (FSO), terrestrial and in space, natural light provides an unmatched amount of energy that can be harvested, yielding the possibility of a bigger power budget for processing. At the same time, in underwater applications, the system will tend to prioritize power-saving features.

The large area receiver panel was tested with natural and artificial light sources. Table 1 shows the electrical output in each scenario and the amount of Lux from each source. Figure 5e shows the measurement setup for energy harvesting testing for direct exposure to sunlight. The maximum power that can be harvested in this situation is 87.33 mW, which is enough to power continuously for all applications but full-speed data transfer with ADC at the maximum sampling rate (power consumption values are presented in Table S1). Nevertheless, a typical sensing application would require much lower data rates than what we can achieve with our system. Thus, the maximum speed of the system can be neglected in favor of power saving.

**Table 1:** Power harvesting under illumination by different sources

Light source	Lux	Open circuit voltage [V]	Short circuit current [mA]
Unilluminated ambient	14	2.57	7.8 × 10^−3^
Incandescent lamp (60 W)	1275	3.82	2.75
Focused Incandescent lamp (75 W)	17780	5.06	21.36
Shaded ambient (indirect sunlight)	1852	3.84	14.10
Direct sunlight	55900	4.10	21.30

The PD configuration of Fig. 3a was used for natural and artificial light energy harvesting. For energy harvesting from artificial light sources, the device can not always be maintained on self-powered capabilities, and an energy storage device must be used, or a charging period must be implemented. Figure 5f shows the charging of a 0.1 F supercapacitor using an artificial source. Two conditions were set: the first with a single PD being used for communication and the second with no PD active for communication. The full charge of the supercapacitor took around 32.2 min for single PD mode and 31 min for no active PD mode. The test was conducted in the dark, with ambient light of 14 lux. Furthermore, the impact of beam incidence angle on the energy harvesting feature is further detailed in Fig. S13 at the SI section Self-powering and tracking under different illumination angles.

Compared to the cube system, the large area receiver has an active area of 900 mm^2^ while each cube plane has 69.3 mm^2^. Under the 60 W incandescent lamp, each cube plane provides an open circuit voltage of 4.66 V and a short circuit current of 2.22 mA. The cube presents a conversion of 0.149 mW/mm^2^, while the large area receiver is 0.12 mW/mm^2^. The PDs of the cube are more efficient; however, its lower active area yields less power than the large area receiver under the same illuminance. Figure S4 in supplementary materials shows the respective dimensions of both receiver panels.

A challenge arises for the self-powered reconfigurable system when harvesting high levels of energy without requiring data processing. If the energy storage device is full, the power fluctuates in the ultra-low power system, and it starts to lose power mostly as heat. The excess of harvest power is compensated by a large increase in the leakage current of the switches. This prevents the MCU from controlling the panel’s reconfiguration. As a solution, the system was programmed to increase its clock speed while idle once the supercapacitor was full and no data needed decoding. This hardware problem is further discussed in the Supplementary Information in Control Loss in Super Harvest Mode section.

## Discussion

The designed device is based on silicon PDs offering a sensitivity covering from 300 to 1100 nm, making it suitable for a wide range of OWC applications, including underwater optical wireless communication, visible light communication, and the 850-nm FSO and even wavelengths used for near earth demonstrations. The use of different wavelengths in OWC has traditionally been focused on maximizing the data rate through wavelength division multiplexing (WDM)^33–36^. Color switching opens the possibility of optimized wavelength use, as it facilitates channel identification and separation, especially in complex networks with overlapping signals, preventing data crosstalk and ensuring signal integrity. For instance, in underwater communication, different wavelengths penetrate water depths differently, making wavelength detection crucial for identifying and routing data from specific underwater sensors. In the specific demonstration of this work, both PDs have better energy harvesting efficiency for the 625–750 nm range. By detecting specific wavelengths within the 625–750 nm range, the device can intelligently disable the PDs dedicated to communication and activate all PDs for energy harvesting. Conversely, the device can enable specific features by switching to a different wavelength range. For example, the device can activate the PD designated for communication by transitioning from the 625–750 nm to the 500–565 nm wavelength range. The internal PGA and DAC allow the creation of an adaptive threshold voltage for different light conditions. When the ambient light is high, the DAC voltage increases and the PGA gain decreases. Moreover, wavelength switching paves the way for standardizing specific wavelengths for custom applications or even implementing the optical frequency hopping^37^. However, a possible approach would be processing the wavelength using multiple receivers, each with a fixed filter. Nevertheless, this would require more processing power, slowing the maximum data rate that can be achieved by frequency hopping.

For a more practical design, we can leverage the use of narrow-band PD with high conversion efficiency in the 4th quadrant of the semiconductor I-V. Photovoltaic laser power converters (PVLPCs) can reach up to 68.9% conversion efficiency for single-junction^38^. Integrating such devices into this work’s proof of concept design would allow for a much higher power budget and better processing capabilities.

Furthermore, an omnidirectional receiver can be helpful in reducing the effect of turbulence-induced beam wandering^39^. Increasing the detection bandwidth of unit PDs comes at the expense of the active area. For instance, high-speed detectors are restricted to a few mm^2^ areas due to the limit imposed by the resistor-capacitor (RC) time constant^40^. Studies showed that the achievable rates could touch the 1 Gbps boundary with GaAs cells illuminated by infrared light vertical cavity surface emitting lasers^41^. Surpassing this challenge can be done by incorporating large field of view (FoV) antennas, particularly fused fiber optic tapers, formed by hundreds of thousands of optical fibers^42^. Such a solution is broadband and will not affect the charging of the device by ambient light. Other solutions to enlarge the FoV of PDs include luminescent solar concentrators or scintillating fibers. Still, both can be wavelength-dependent and unstable for use for an extended time^43^. An omnidirectional receiver with EH capability can be very interesting for harsh environment operations and can be installed on sensors requiring continuous commands. Another potential use case of the omnidirectional receiver is in CubeSats and outer space applications (see Fig. 1), mainly because the device can harvest energy from the sun while orbiting. Other applications of the self-powered omnidirectional receiver involve visible light communication (VLC)^44^ and underwater wireless optical communication (UWOC)^45^.

The current design of the system provides six receiver planes in the form of a cube, and one of the receiver planes is removable to provide access to the switching matrix boards through a flexible flat ribbon cable (FFC). A stack-up solution is used to multiplex all the inputs from all planes (Fig. S4a).

Mechanically moving the PD to the individual faces of the cubes can be challenging due to the inner circuitry. A possible system improvement is the fabrication of flexible substrates using micro-PD arrays for truly omnidirectional application. The arrays can be manufactured in a single flexible PCB sheet and be folded using an origami-like approach. Using micro-PD arrays would also add the benefit of having higher bandwidth than large areas PDs.

This paper reports a major step towards developing energy-efficient omnidirectional optical communications systems. By leveraging the reconfiguration of the PD in its fundamental photoconductive and photovoltaic mode, we can achieve realistic higher data rates while harvesting the maximum power from the PD. It is worth noting that the development of miniaturized hardware for OWC/UWOC brings complexity resulting in bandwidth losses, mostly due to the switch circuitry. As one of the first devices to encompass the entire SLIPT circuitry interface and decoding, the maximum achieved data rate of 85.2 Mbps can only be displayed in real-time using an oscilloscope and be processed offline, as the hardware is not capable of processing and decoding the large volume of data. Nevertheless, the gross rate of 5 Mbps from a completely self-powered SLIPT system is the highest reported, both in free space and underwater. Furthermore, the introduction of a wavelength-based SLIPT paves the way for standardization and optimization of SLIPT-based systems, in which we demonstrated a hardware-level implementation that can be designed in-chip to also achieve great energy efficiency.

## Materials and methods

### Main board circuit design

The system is composed of two MCUs (STM32L4R9 and CY8C5888), a switch matrix composed of 14 analog switches (Texas Instruments TS3A24159), a Quad-SPI flash memory (25Q128jvsq), an SD card slot and an external TIA from Texas Instruments (OPA380). The system utilizes its two MCUs with distinct roles, which can work in parallel for optimal performance or only one active to preserve power. The main MCU is an STM32L4R9 ultra-low-power, high-performance 32-bit microcontroller with Arm® Cortex®-M4 core, DSP, and floating-point unit (FPU). In this project, this MCU digitizes the analog voltage output from the TIA and stores it in the SD card using the ping-pong buffer technique with flash memory. The second MCU is the CY8C5888 PSoC from Infineon Technologies AG. The PSoC interfaces with the wavelength detector, measures the signal strength using its internal TIAs, and controls the switch matrix. The PSoC and the STM32 share a UART bus and GPIOs used for communication between both MCUs.The circuit was designed and simulated using the Proteus Design Suite from Labcenter Electronics. The signal traces were shielded using a stitching technique, and ground splitting was used to separate digital from analog zones. The PCB was manufactured and assembled using standard FR-4 base material with 1.6 mm thickness and 1 oz outer copper weight. The internal circuitry of the PSoC was designed using PSoC Creator 4.4 software. The firmware of both MCUs was done in C + +, using STMCube Integrated Design Environment (IDE) for the STM32 and PSoC Creator IDE for the PSoC.

For color detection, two hardware designs were implemented in the PSoC. The first one makes use of a TIA followed by an ADC. The CPU core processes the digitized signal from the color detectors through a custom C + + code. The code averages the ambient light automatically, taking it as a baseline, using the averaged value as a threshold for detecting sudden increases in luminosity due to light beams being directly pointed at the device. The different color channels are selected using an analog multiplexer at the negative input of the TIA. The data on which color is dominant is sent from the PSoC to the STM32 through the UART protocol. The second design dispenses the use of the PSoC CPU core, as all the detection is done via hardware, and the color detection is output in binary and interpreted by the STM32. The section Color-based SLIPT presents the second design in detail. Fig. S4 shows the main board device.

An off-the-shelf ultra-low-power boost converter with a battery management system (Texas Instruments BQ25504 EVM) controls the energy harvesting process. The BQ25504 is specifically made to efficiently collect and control the power generated from various DC sources, and it has an integrated dynamic maximum power point tracking for optimal energy extraction. A 5 V 0.1-F supercapacitor was used as an energy storage device (EATON PB B3-129).

### Receiver panel circuit design

The large receiver panel comprises nine red-enhanced high-performance silicon PD (Luna Optoelectronics sd445-14-21-305) enclosed in hermetically sealed ceramic packages. Each PD has an active area of 1 cm^2^ in a 2.24 cm^2^ package. The PDs were arranged in a 3 × 3 matrix with minimal spacing between the packages. Surrounding the PD matrix are three RGB color sensors (Kingbright APS5130PD7C-P22) placed to detect the specific light colors reaching the receiver panel. The color sensors utilize three integral color filters and silicon PD with peak intensities at blue (470 nm), green (550 nm), and red (620 nm) wavelengths. A 90° angle header with a 2.54 mm pitch was selected as a connection. The printed circuit board (PCB) is manufactured with a standard FR-4 base material of 1.6 mm thickness and 1 oz outer copper weight. The part components were purchased from Digi-Key Electronics, and the assembly was performed in-house. Fig. S4 shows the receiver panel.

The small receiver panels are built in a 2 x 2 cm squared-shaped PCB containing 9 equally spaced PDB-C171SM Advanced Photonix PD on its top layer and an FFC connector on its bottom layer. The multi-plane, or cube-shaped, receiver was built using 6 identical squared-shaped PCBs manufactured with standard FR-4 base material of 1.6 mm thickness and 1-oz outer copper weight. The small receiver panel boards were designed and assembled in-house, and the components were purchased from Digi-Key Electronics.

### Wavelength-based SLIPT

The color detector from the receiver panel is connected to the PSoC, where a hardware design was implemented to facilitate color detection and provide a digital output. The design process was carried out using PSoC Creator 4.4 software. The three output voltages from the color sensor are individually amplified by three programmable gain amplifiers (PGA) that operate in parallel. The system can handle the different color signals simultaneously and independently by using multiple PGAs, each corresponding to a specific color channel. This parallel processing enables efficient color detection and analysis. The amplified voltage from each PGA is separately compared against a single threshold voltage created by a digital-to-analog voltage converter (DAC). The three comparator outputs feed the inputs of a lookup table (LUT) that provides the two-bit results. The LUT is configured to provide an output only when a single color is predominant. Both the threshold voltages from the DAC and the gain of the PGA are adjustable to accommodate varying ambient light conditions.

At the transmitter side, two lasers were used: a 450 nm (Osram PL-TB450B) and a 658 nm (Mitsubishi Laser Diodes ML101J23). The transmitter also comprised a high-power LED with a peak intensity of 530 nm. Each laser was connected to the power source and signal generator through a bias tee (Picosecond Pulse Labs 5543-223). The bias voltages were generated by two dual-output DC power supplies (Agilent E3649A). The OFDM signal was generated using a waveform generator (Keysight 33600 A). DC measurements were performed using a source meter (Keithley 2450). A mixed signal oscilloscope (Tektronix MSO 4104) was used to measure the three analog outputs at the PGA stage. A digital storage oscilloscope (Agilent DSO-X 2024 A) was used to measure the system’s digital output.

### Power harvesting

A 60 W incandescent lamp (Reflector R80) was placed indoors at 20 cm from the device to evaluate the energy harvesting capability using an artificial light source. The experiment was conducted in a dark room with only the lamp turned on. The light intensity was measured using a light meter (Extech 401025) positioned beside the receiver facing the lamp. One PD was configured as an information receiver in the system, while the remaining PDs were set as energy harvesters. The device was then positioned in two locations: inside a room with open windows to measure under diffuse sunlight and outside under direct sunlight. The light intensity in both places was also measured using the light meter. The system’s open circuit voltage and short circuit current were measured using a digital multimeter (Keysight 34470 A). The collected data were plotted using a custom Python code.

### Coding/decoding information

A pseudo-random binary sequence (PRBS) was generated and modulated using a quadrature amplitude modulation (QAM) scheme. A Hermitian symmetry was applied before the inverse Fast Fourier transform (IFFT) to provide real values. The used bandwidth was between 0.015 and 1.5 MHz, and the size of the IFFT was 1024. A cyclic prefix length of ten was used to limit intersymbol interference. The minimum allowed SNR per subcarrier was 3 dB. The parallel sequence was converted to a serial sequence and then sent by the arbitrary waveform generator to a laser through a bias tee. At the receiver, the signal was down-sampled and synchronized. The serial sequence was then converted to parallel. The CP was removed, and a Fast Fourier Transform (FFT) was applied. By removing the symbols of the Hermitian symmetry, the symbols were extracted. The symbols were then demodulated and converted to a serial stream of bits, which was compared to the transmitted signal to determine the BER. The gross data rate, *GDR*, is calculated using the following formula:

1 $${GDR}=\frac{{f}_{{AWG}}}{{N}_{{FFT}}+{N}_{{Cp}}}\mathop{\sum }\limits_{i=1}^{{N}_{{SC}}}{\log }_{2}\left({M}_{i}\right)$$

with *N*_SC_ = 500 is the number of subcarriers. *M*_*i*_ = 2^*b*^ is the QAM order of the i^*th*^ subcarrier, and *b* is the number of bits allocated to that subcarrier. The net data rate is calculated by accounting for the training symbols used for channel estimation and synchronization and the 7% FEC overhead. Figures S6 and S7 show the coding/decoding information setup for both the main receiver panel and the cube system. Each subcarrier is assigned a spectral efficiency based on the calculated signal-to-noise ratio (SNR). The SNR is estimated by sending uniform 4-QAM OFDM symbols and calculating the error vector magnitude (EVM). Based on that, the maximum achievable spectral efficiency is estimated as log_2_(1 + SNR).

### Single PD selection and quadrant control

For single PD selection, the system is configured to reverse bias one of the PDs from the matrix. At the same time, the remaining PDs are connected in series, as seen in Fig. 3a. The output of the in-series PDs is connected to the energy harvesting circuit. The switching and biasing circuit transfer function *A*(*dB*) is given by (2) and its circuit representation for each PD is given in Fig. S10.

2 $$A\left({bB}\right)=10\log \left[{\omega }^{2}{({R}_{{on}}{C}_{{DS}})}^{2}+1\right]+20\log \left[\frac{{R}_{{load}}}{{R}_{{load}}{R}_{{on}}}\right]-10\log {\omega }^{2}\left[{\left(\frac{{R}_{{load}}}{{R}_{{load}}{R}_{{on}}}\right)}^{2}{({C}_{{load}}+{C}_{D}+{C}_{{DS}})}^{2}+1)\right]$$

where *C*_*DS*_ is the internal drain to source capacitance of the switch, *C*_*D*_ is the drain to ground capacitance, and *C*_*load*_ and *R*_*load*_ are the capacitance of the negative input connection of the TIA seen by the output of the switch. The parasitic *C*_*load*_ capacitance is highly dependent on the traces in the PCB. *R*_*on*_ is the internal resistance of the switch when on. As (2) only represents the switching and biasing circuit, we do not consider the input and shunt resistance of the PD, which are dependent on the light and voltage bias. For the quadrant control, we divided the PD matrix into ten different quadrants, as presented in Fig. 4d. The quadrant is formed with the 4 PDs next to each other or the row and column of the matrix. When the quadrant is in harvesting mode, more than one quadrant can overlap each other. In the information decoding mode, the PDs are isolated, while in the harvesting mode, the PDs are connected in parallel. Even when isolated, there is channel-to-channel crosstalk. Fig. S11 shows the equivalent circuit model for the crosstalk in the quadrant mode.

### Supplementary information

Final Supplemental Information

## References

[1] Krikidis, I. et al. Simultaneous wireless information and power transfer in modern communication systems. *IEEE Commun. Mag.* **52**, 104–110 (2014). 10.1109/MCOM.2014.6957150

[2] Perera, T. D. P. et al. Simultaneous wireless information and power transfer (SWIPT): Recent advances and future challenges. *IEEE Commun. Surv. Tutor.* **20**, 264–302 (2018). 10.1109/COMST.2017.2783901

[3] Thomas, S. Connecting with wireless power transfer. *Nat. Electron.* **6**, 106 (2023). 10.1038/s41928-023-00933-z

[4] Ozaki, T. et al. A wireless radiofrequency-powered insect-scale flapping-wing aerial vehicle. *Nat. Electron.* **4**, 845–852 (2021). 10.1038/s41928-021-00669-8

[5] Song, M. Z. et al. Wireless power transfer based on novel physical concepts. *Nat. Electron.* **4**, 707–716 (2021). 10.1038/s41928-021-00658-x

[6] Bakytbekov, A. et al. Fully printed 3D cube-shaped multiband fractal rectenna for ambient RF energy harvesting. *Nano Energy* **53**, 587–595 (2018). 10.1016/j.nanoen.2018.09.022

[7] Lee, J. et al. Neural recording and stimulation using wireless networks of microimplants. *Nat. Electron.* **4**, 604–614 (2021). 10.1038/s41928-021-00631-8

[8] Wang, X. et al. High-performance cost efficient simultaneous wireless information and power transfers deploying jointly modulated amplifying programmable metasurface. *Nat. Commun.* **14**, 6002 (2023). 37752144 10.1038/s41467-023-41763-z PMC10522703

[9] Fakidis, J. et al. Indoor optical wireless power transfer to small cells at nighttime. *J. Lightwave Technol.* **34**, 3236–3258 (2016). 10.1109/JLT.2016.2555883

[10] De Oliveira Filho, J. I. et al. Self-powered weather station for remote areas and difficult-access locations. *Opt. Express* **30**, 2668–2679 (2022). 35209402 10.1364/OE.441983

[11] Tavakkolnia, I. et al. Organic photovoltaics for simultaneous energy harvesting and high-speed MIMO optical wireless communications. *Light Sci. Appl.* **10**, 41 (2021). 33623027 10.1038/s41377-021-00487-9 PMC7902835

[12] De Oliveira Filho, J. I. et al. Simultaneous lightwave and power transfer for internet of things devices. *Energies* **15**, 2814 (2022). 10.3390/en15082814

[13] de Oliveira Filho, J. I. et al. Toward self-powered internet of underwater things devices. *IEEE Commun. Mag.* **58**, 68–73 (2020). 10.1109/MCOM.001.1900413

[14] Kim, S. M. & Won, J. S. Simultaneous reception of visible light communication and optical energy using a solar cell receiver. 2013 International Conference on ICT Convergence (ICTC). Jeju: IEEE, 2013, 896–897.

[15] Wang, Z. X. et al. On the design of a solar-panel receiver for optical wireless communications with simultaneous energy harvesting. *IEEE J. Sel. Areas Commun.* **33**, 1612–1623 (2015). 10.1109/JSAC.2015.2391811

[16] Zhang, S. Y. et al. Organic solar cells as high-speed data detectors for visible light communication. *Optica* **2**, 607–610 (2015). 10.1364/OPTICA.2.000607

[17] Fakidis, J. Videv, S. Helmers, H. & Haas, H. 0.5-Gb/s OFDM-based laser data and power transfer using a GaAs photovoltaic cell. 2018 IEEE Photonics Conference (IPC). Reston, VA, USA, 1–4. 10.1109/IPCon.2018.8527236 (2018).

[18] Mica, N. A. et al. Triple-cation perovskite solar cells for visible light communications. *Photonics Res.* **8**, A16–A24 (2020). 10.1364/PRJ.393647

[19] Baiden G. & Barroso A. R. Inventors; Penguin Automated Systems Inc, assignee. Omnidirectional optical wireless communications receiver and system. United States patent US 10,204,514 (2019).

[20] Cincotta, S. et al. High angular resolution visible light positioning using a quadrant photodiode angular diversity aperture receiver (QADA). *Opt. Express* **26**, 9230–9242 (2018). 29715877 10.1364/OE.26.009230

[21] Kaushal, H. & Kaddoum, G. Optical communication in space: Challenges and mitigation techniques. *IEEE Commun. Surv. Tutor.* **19**, 57–96 (2017). 10.1109/COMST.2016.2603518

[22] Bergeron, H. et al. Femtosecond time synchronization of optical clocks off of a flying quadcopter. *Nat. Commun.* **10**, 1819 (2019). 31000702 10.1038/s41467-019-09768-9 PMC6472402

[23] Walsh, S. M. et al. Demonstration of 100 Gbps coherent free-space optical communications at LEO tracking rates. *Sci. Rep.* **12**, 18345 (2022). 36316353 10.1038/s41598-022-22027-0 PMC9622843

[24] Zhao, M. M. et al. Long-reach underwater wireless optical communication with relaxed link alignment enabled by optical combination and arrayed sensitive receivers. *Opt. Express* **28**, 34450–34460 (2020). 33182914 10.1364/OE.410026

[25] Zeng, L. B. et al. High data rate multiple input multiple output (MIMO) optical wireless communications using white LED lighting. *IEEE J. Sel. Areas Commun.* **27**, 1654–1662 (2009). 10.1109/JSAC.2009.091215

[26] Peyronel, T. et al. Luminescent detector for free-space optical communication. *Optica* **3**, 787–792 (2016). 10.1364/OPTICA.3.000787

[27] Manousiadis, P. P. et al. Wide field-of-view fluorescent antenna for visible light communications beyond the étendue limit. *Optica* **3**, 702–706 (2016). 10.1364/OPTICA.3.000702

[28] Kang, C. H. et al. Ultraviolet-to-blue color-converting scintillating-fibers photoreceiver for 375-nm laser-based underwater wireless optical communication. *Opt. Express* **27**, 30450–30461 (2019). 31684293 10.1364/OE.27.030450

[29] Sarbazi, E. et al. Imaging angle diversity receiver design for 6G optical wireless communications: performance tradeoffs and optimisation. GLOBECOM 2023-2023 IEEE Global Communications Conference. Kuala Lumpur: IEEE, 5555–5560 (2023).

[30] Sarbazi, E. et al. Design and optimization of high-speed receivers for 6G optical wireless networks. *IEEE Trans. Commun.* **72**, 971–990 (2024). 10.1109/TCOMM.2023.3328265

[31] Khalighi, M. A. & Uysal, M. Survey on free space optical communication: A communication theory perspective. *IEEE Commun. Surv. Tutor.* **16**, 2231–2258 (2014). 10.1109/COMST.2014.2329501

[32] Yang, Z. Y. et al. High performance mechano-optoelectronic molecular switch. *Nat. Commun.* **14**, 5639 (2023). 37704605 10.1038/s41467-023-41433-0 PMC10499996

[33] Zou, K. H. et al. High-capacity free-space optical communications using wavelength- and mode-division-multiplexing in the mid-infrared region. *Nat. Commun.* **13**, 7662 (2022). 36496483 10.1038/s41467-022-35327-w PMC9741622

[34] Wang, Y. G. et al. 8-Gb/s RGBY LED-based WDM VLC system employing high-order CAP modulation and hybrid post equalizer. *IEEE Photonics J.* **7**, 7904507 (2015).

[35] Cui, L. et al. Analysis of the multichannel WDM-VLC communication system. *J. Lightwave Technol.* **34**, 5627–5634 (2016). 10.1109/JLT.2016.2623759

[36] Gutema, T. Z., Haas, H. & Popoola, W. O. WDM based 10.8 Gbps visible light communication with probabilistic shaping. *J. Lightwave Technol.* **40**, 5062–5069 (2022). 10.1109/JLT.2022.3175575

[37] Wang, S. L. et al. A novel optical frequency-hopping scheme for secure WDM optical communications. *IEEE Photonics J.* **7**, 7201108 (2015). 10.1109/JPHOT.2015.2429635

[38] Algora, C. et al. Beaming power: Photovoltaic laser power converters for power-by-light. *Joule* **6**, 340–368 (2022). 10.1016/j.joule.2021.11.014

[39] Trichili, A. et al. Roadmap to free space optics. *J. Optical Soc. Am. B* **37**, A184–A201 (2020). 10.1364/JOSAB.399168

[40] Zhao, Z. P. et al. High-speed photodetectors in optical communication system. *J. Semiconductors* **38**, 121001 (2017). 10.1088/1674-4926/38/12/121001

[41] Fakidis, J., Helmers, H. & Haas, H. Simultaneous wireless data and power transfer for a 1-Gb/s GaAs VCSEL and photovoltaic link. *IEEE Photonics Technol. Lett.* **32**, 1277–1280 (2020). 10.1109/LPT.2020.3018960

[42] Alkhazragi, O. et al. Wide-field-of-view optical detectors using fused fiber-optic tapers. *Opt. Lett.* **46**, 1916–1919 (2021). 33857103 10.1364/OL.423437

[43] Alkhazragi, O. et al. Toward wide-field-of-view and large area optical detectors for high-speed optical wireless communication. *IEEE Commun. Mag.* **61**, 162–167 (2023). 10.1109/MCOM.001.2200804

[44] Shin, W. H. et al. Self-reverse-biased solar panel optical receiver for simultaneous visible light communication and energy harvesting. *Opt. Express* **24**, A1300–A1305 (2016). 27828517 10.1364/OE.24.0A1300

[45] Uysal, M. et al. SLIPT for underwater visible light communications: Performance analysis and optimization. *IEEE Trans. Wirel. Commun.* **20**, 6715–6728 (2021). 10.1109/TWC.2021.3076159

