# Supplementary material for: Reconfigurable MIMO-based self-powered battery-less light communication system
Source: Light Sci Appl. 2024 Aug 28;13:218. doi: 10.1038/s41377-024-01566-3 (PMC11358267; doi:10.1038/s41377-024-01566-3)
Supplement: Supplementary file 1 — Final Supplemental Information [file 41377_2024_1566_MOESM1_ESM.docx]

**Supplementary Information for**

**Reconfigurable MIMO-based Self-powered** **Battery-less Light Communication System**

# Jose Ilton De Oliveira Filho^1,2,*^, Abderrahmen Trichili^1^, Omar Alkhazragi^1^, Mohamed-Slim Alouini^1^, Boon S. Ooi^1^, and Khaled Nabil Salama^1^

^1^Computer, Electrical and Mathematical Sciences & Engineering, King Abdullah University of Science and Technology, Thuwal, Saudi Arabia.

^2^NEOM Institute for Ocean Science and Solutions, Neom, Saudi Arabia.

[^*^jose.deoliveirafilho@kaust.edu.sa](mailto:*jose.deoliveirafilho@kaust.edu.sa)


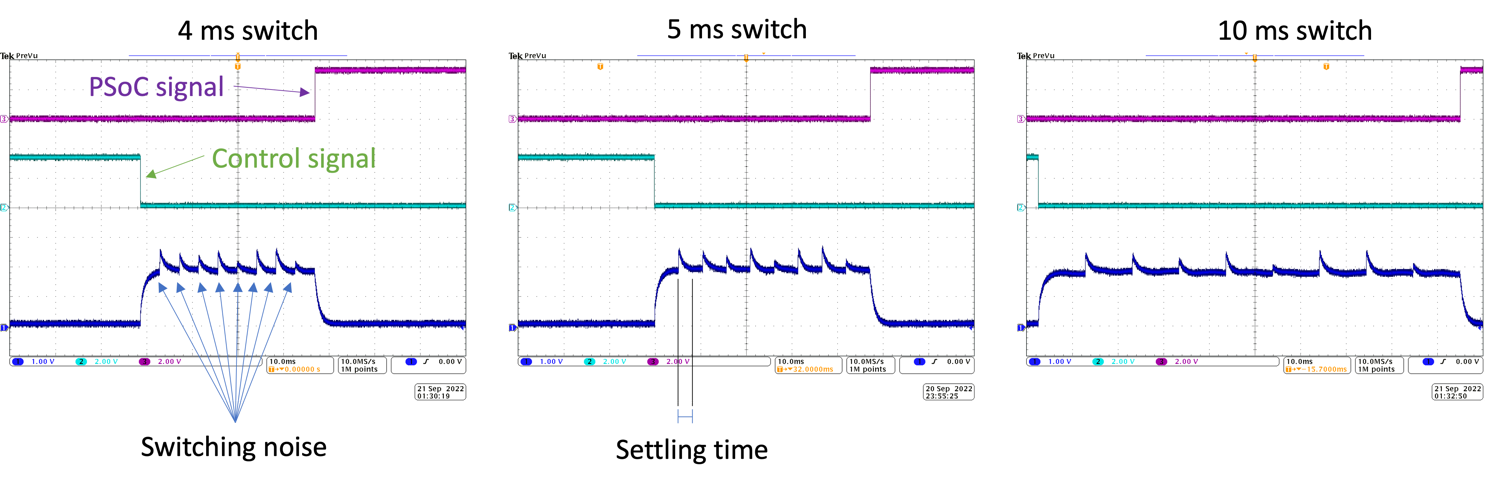


**Figure S1.** Signals indicate the switching between all the PDs with three distinct switching times. The green signal indicates the pulse from the main MCU to the PSoC to start the sweeping of all PDs to find the best SNR. The purple signal indicates the PSoC’s output, informing the main MCU that the sweep is done. The blue signal indicates the voltage output of the receiver panel.


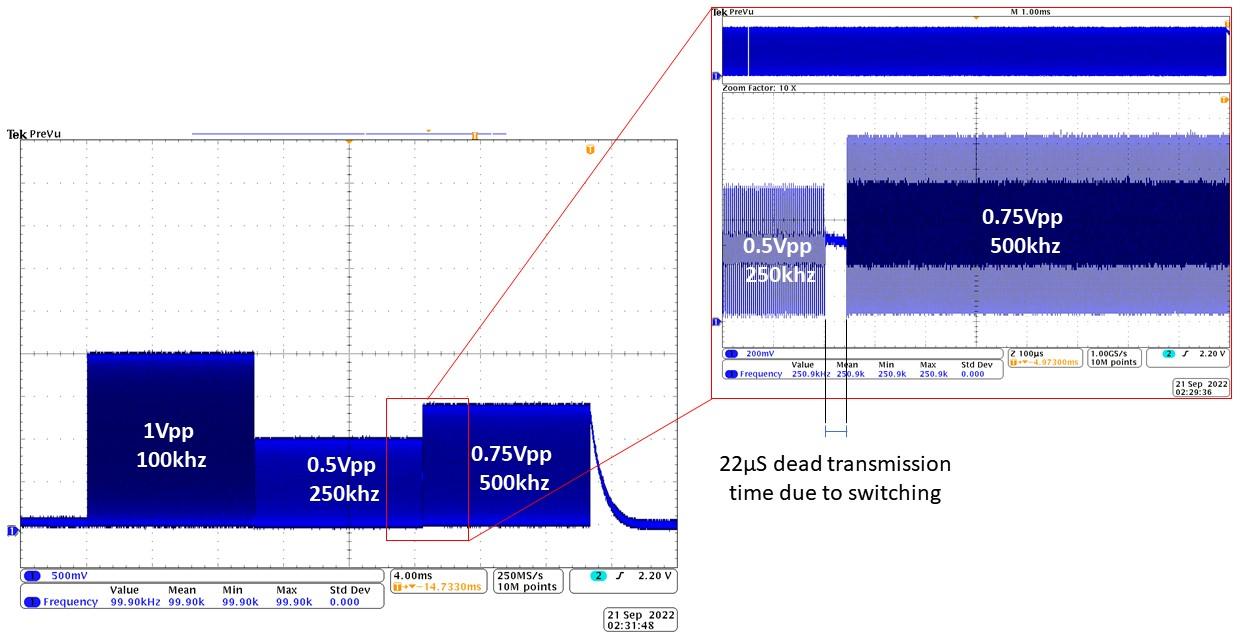


**Figure S2.** Oscillograms of a single output being multiplexed from three distinct signals feed to three distinct PD. The switching from each PD takes approximately 22 *µs*.

**Table S1.** Power consumption values at different tasks

| Activity | Voltage [V] | Current[mA] | Power [mW] |
| --- | --- | --- | --- |
| Both MCU and PSoC in sleep (TIA off) | 3.3 | 0.9 | 2.97 |
| SNR searching (MCU sleep, PSoC On) | 3.3 | 5.2 | 17.6 |
| Select PD and hold reverse biased (MCU On, PSoC sleep) | 3.3 | 2.7 | 8.91 |
| Wavelength and transmission detection (MCU On, PSoC On) | 3.3 | 3.4 | 11.22 |
| PD reverse biased with TIA (MCU On, PSoC sleep) | 3.3 | 10.1 | 33.33 |
| PD reverse biased with external TIA saving data at SD card | 3.3 | 51.9 | 171.27 |


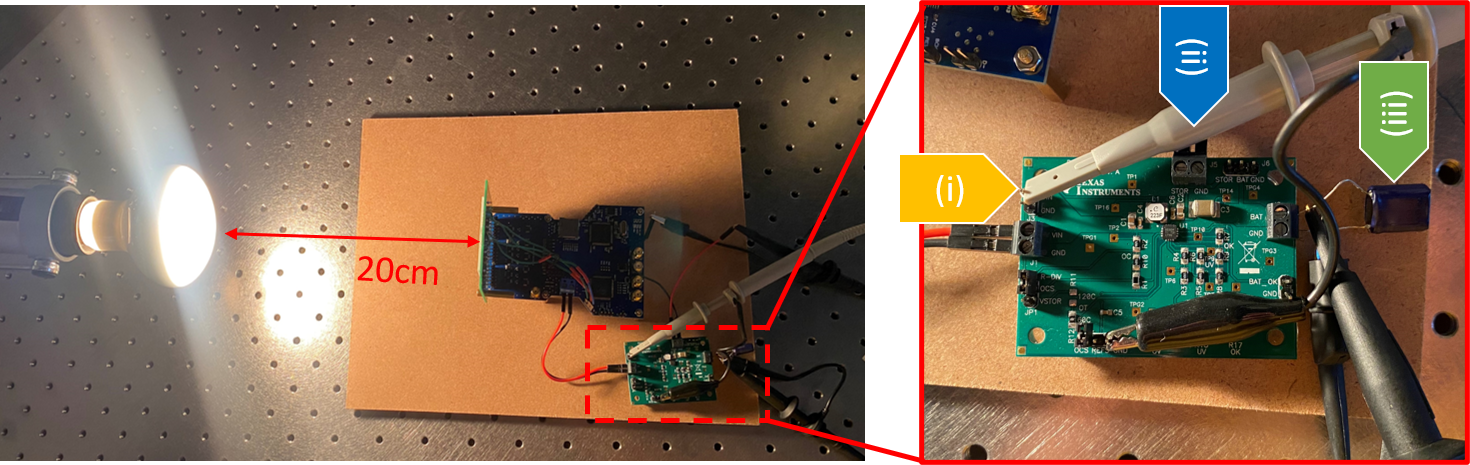


**Figure S3.** Setup for energy harvesting from artificial light sources with (i) input at the receiver panel voltage, (ii) output to load/receiver system, and (iii) supercapacitor.


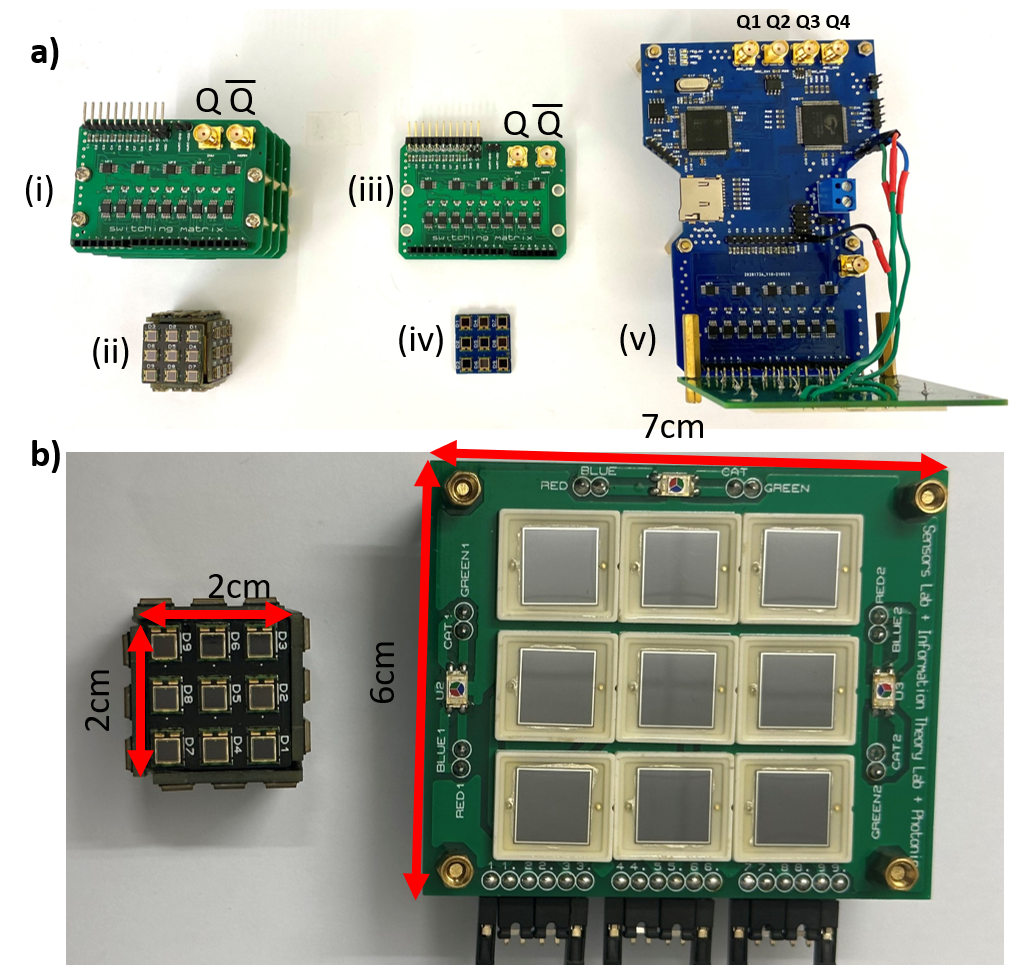


**Figure S4.** a) Hardware modules with (i) stack-up of switching matrices for (ii) cube-shaped receiver, (iii) single switching matrix for (iv) single plane receiver, and (v) full integrated system for controlling, decoding, and harvesting energy from light beams. b) Large area receiver for maximizing power harvesting and small area receiver for maximizing data transfer.


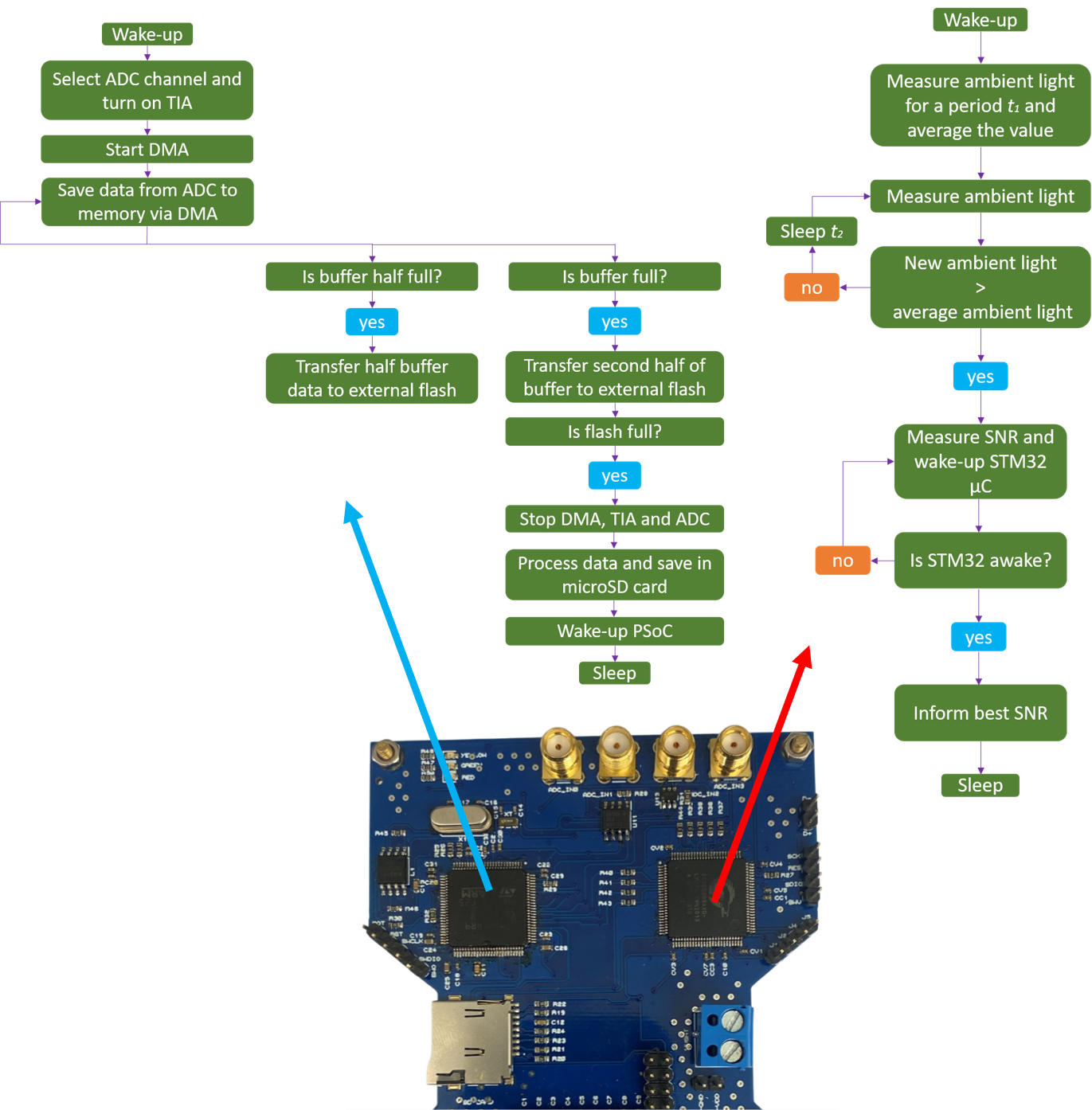


**Figure S5.** Main firmware of STM32 and PSoC devices.


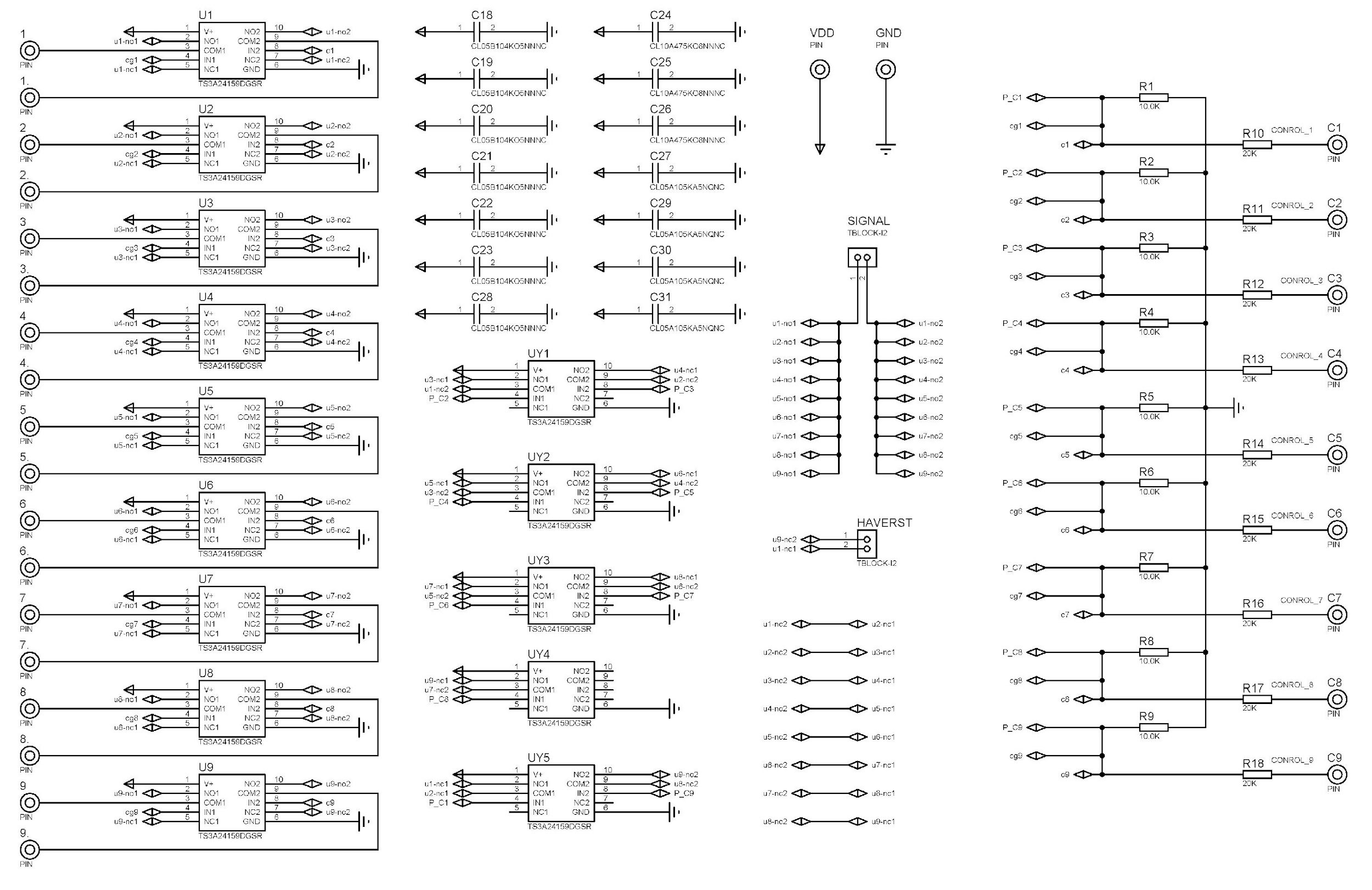


**Figure S6.** Switching matrix circuit.


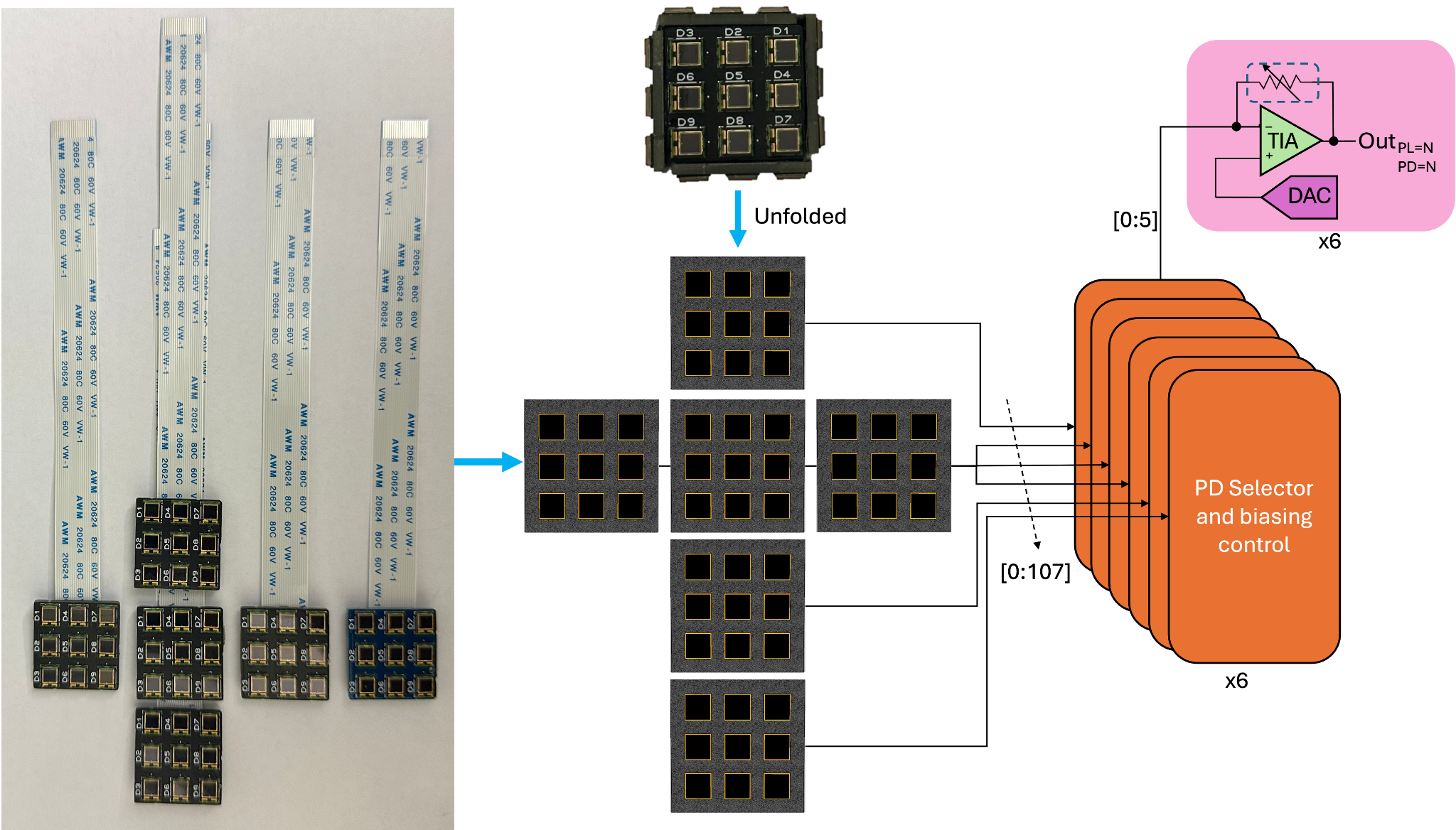


**Figure S7.** Circuit diagram of 3D-plane receiver interface.


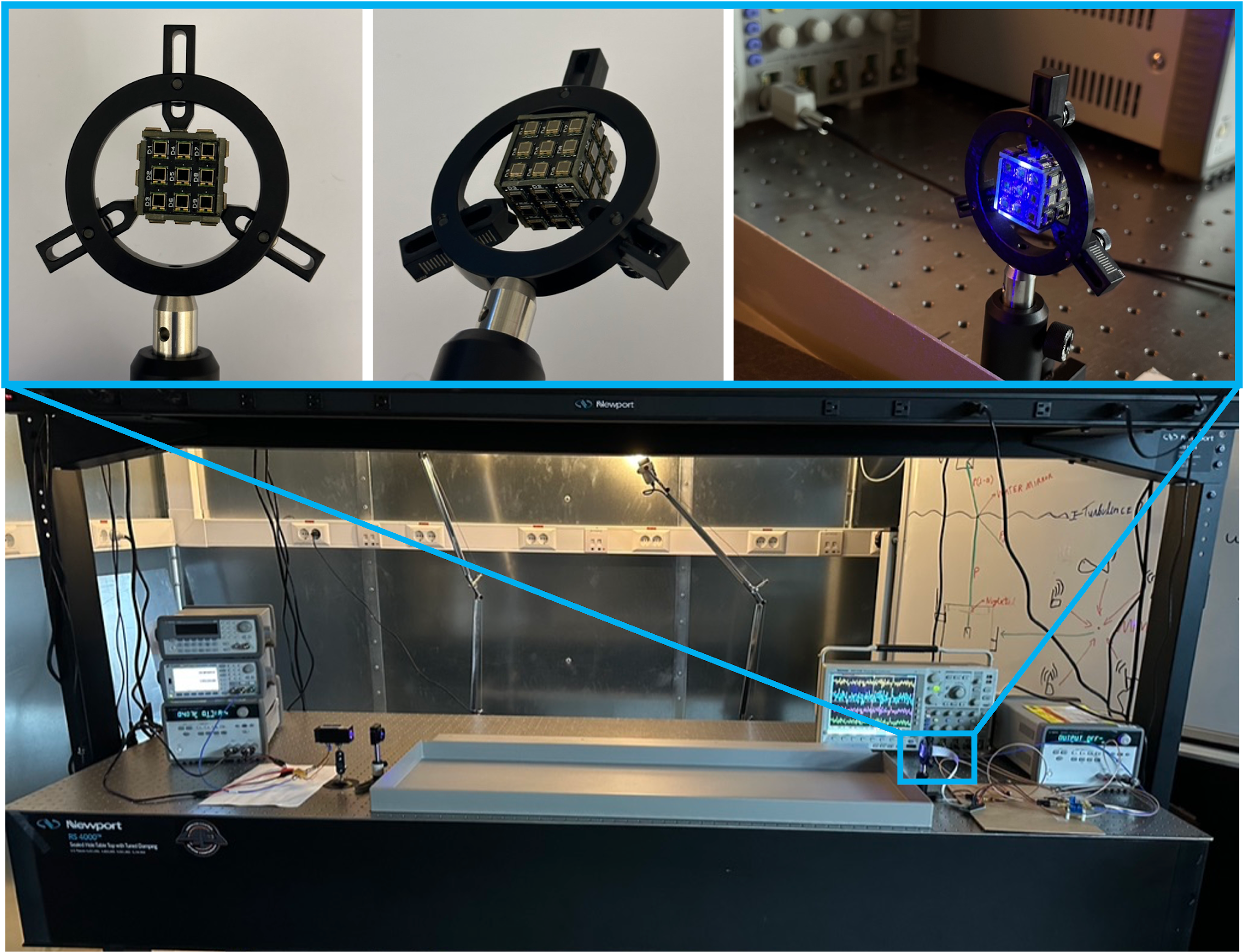


**Figure S8.** Cube system setup.


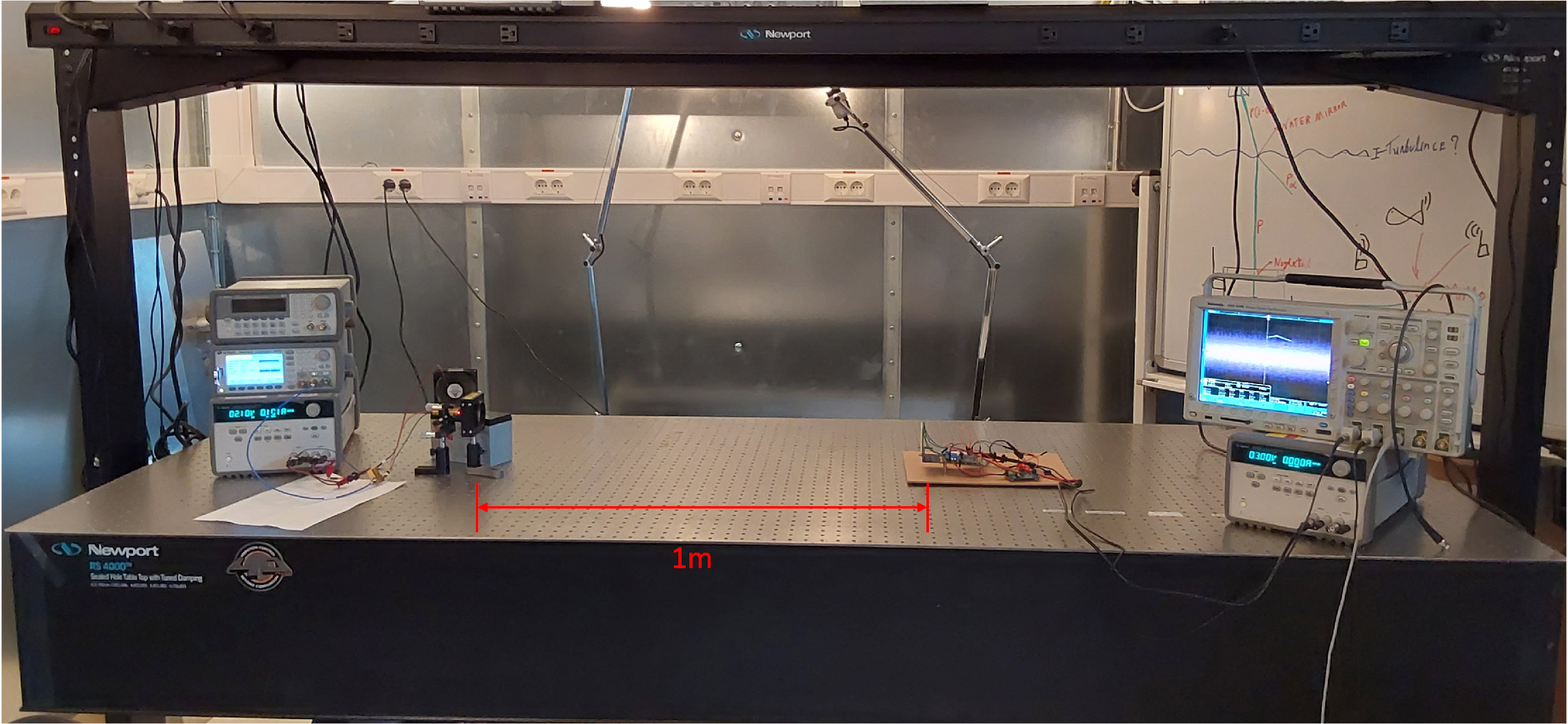


**Figure S9.** Information transfer setup.


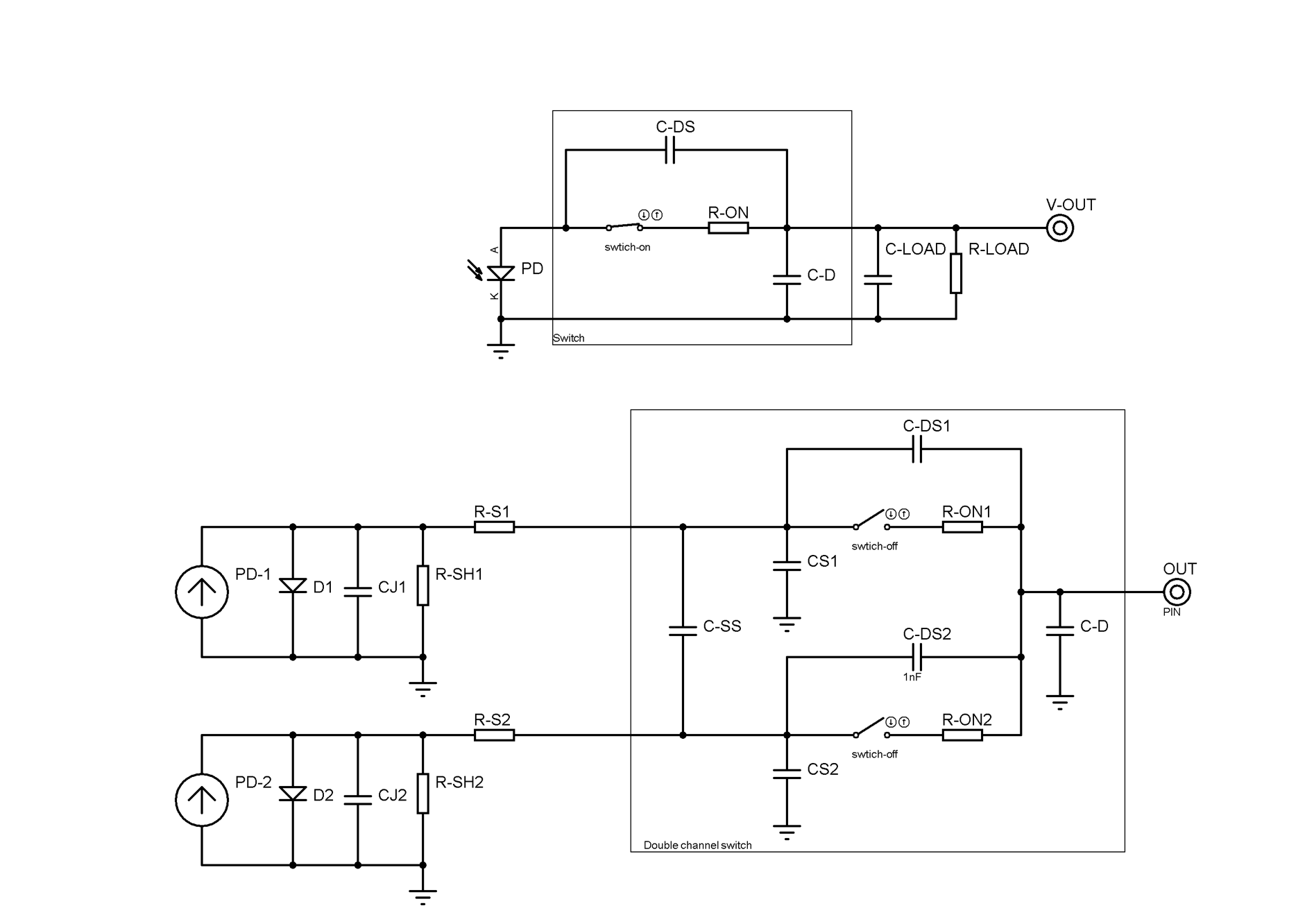


**Figure S10.** Equivalent circuit model for single PD section.


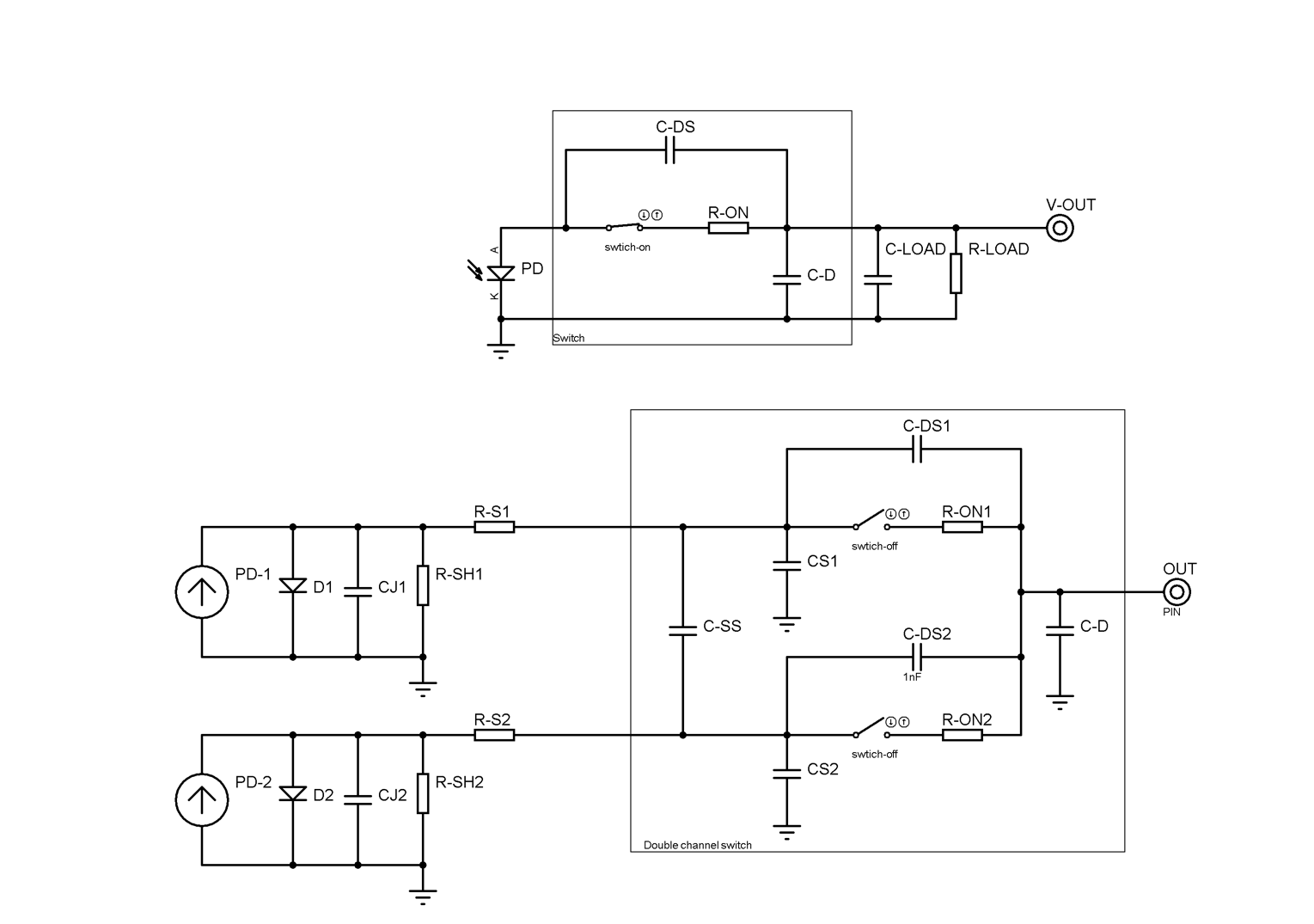


**Figure S11.** Equivalent circuit model for the crosstalk in quadrant model.

**Control loss in super harvest mode.**

Once a strong beam is directly over all PDs, the receiver panels produce twice as much needed for the system to fully operate. Specifically, when connecting the receiver panel in series and exposing the generating voltage that exceeds the capacity of the switches. If the energy storage device is full, the power fluctuates in the ultra-low power system and starts to be lost mostly as heat. Replacing the switches with higher voltage-tolerant ones would increase power consumption and strain the system’s power budget when no strong light source is used. Therefore, keeping the low-power switches is crucial. This increases the current draw, keeping the switches stable. A secondary solution is to disconnect a few PDs from the series, lowering the harvested voltage. However, the current system doesn’t employ voltage measurements at the input for all PDs, as this would be impractical in large matrix designers and would significantly increase the circuit complexity and possible leakage current for signal and harvesting.

**Self-powering and tracking under different illumination angles**

A 450-nm (Osram PL-TB450B) was positioned 50 cm from the receiver plane in a direct line of sight. The receiver plane was rotated from 0 to 90° at its axis, as shown in Figure S13. The angle was measured with a protractor with reference at the middle row of the PD matrix. A pseudo-random binary sequence (PRBS) was generated and modulated using a quadrature amplitude modulation (QAM) scheme. A Hermitian symmetry was applied before the inverse Fast Fourier transform (IFFT) to provide real values. The signal was transmitted, and the SNR at the receiver was calculated using the known PRBS. The SNR of the central PD was compared with the other 8 adjacent PDs. Once the beam wanders at an angle, the beam reaches more than one PD, creating the pattern of shifting the SNR from the center PD to the adjacent PD corresponding to the orientation in which the PD is wandering. The orientation of the laser beam is determined by the active PD row. Figure S13. b) shows the SNR of the middle row of the PD matrix. The angle can be calculated by the SNR ratio between the PDs. Once at an angle, the SNR shift is smoother than when the beam wanders in a direct line of sight, as shown in our results (Figure 4). If the beam wandering speed is below the maximum SNR scan of 43ms, the laser can be successfully tracked. Furthermore, suppose the beam is too misaligned, and the minimum SNR is only detected by the border PDs. In that case, our tracking methodology can not provide useful information regarding the orientation and tracking of the beam. Nevertheless, the beam tracking serves the only purpose of providing the best SNR possible; once the best SNR of all PDs is detected, the system will switch to the best PD and keep switching automatically or switch only once the communication link is lost.


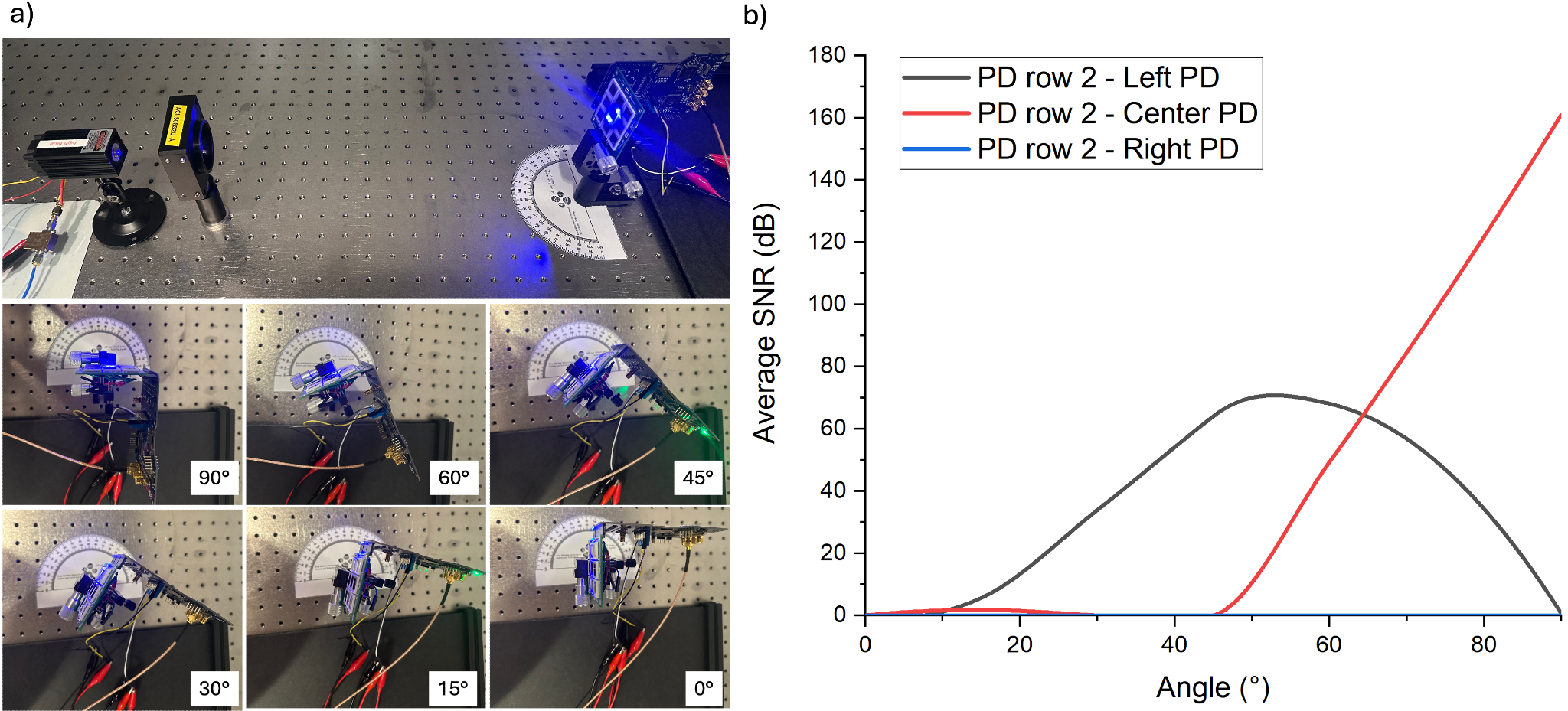


Figure S12. Laser beam measurement under different angles. a) Laser beam setup with incident angles of 90°, 60°, 45°, 30°, 15°, and 0°. b) Average SNR of signal received at the middle row. Only the middle row PD matrix presents SNR higher than 0.

A 75-W spotlight was positioned 10 cm from the receiver plane, in direct line of sight. The receiver plane was rotated from 0 to 180° at its axis. The angle was measured using a protractor with reference at the middle row of the PD matrix. The receiver system and switch matrix were externally powered to accurately measure the generated power. The power generated with respect to each angle is plotted in Figure S13. b). After the power measurement, the power consumption in self-powered batteryless mode was measured, and it is also plotted in Figure S13. b). Each self-power mode is represented at “P” from 1 to 4, corresponding to P1: Both MCU and PSoC in sleep; P2: Select PD and hold reverse bias; P3: Wavelength and transmission detection; P4: SNR searching. As a result, for an angle lower than 20°, our device can not harvest enough light (considering a source of X lux) to power itself in continuous operation. However, for greater angles (~ >20°) the device can sustain operation without battery in power modes P1-4.

The device is not able to hold self-powering mode without harvesting period for P5 (photodetector in reverse bias with PSoC internal transimpedance amplifier enabled converting the signal) and P6 (photodetector in reverse bias with external transimpedance amplifier at SD card saving at higher data speeds). Nevertheless, P5-6 can be achieved using a storage device, for example, a supercapacitor, with prolonged harvesting time.


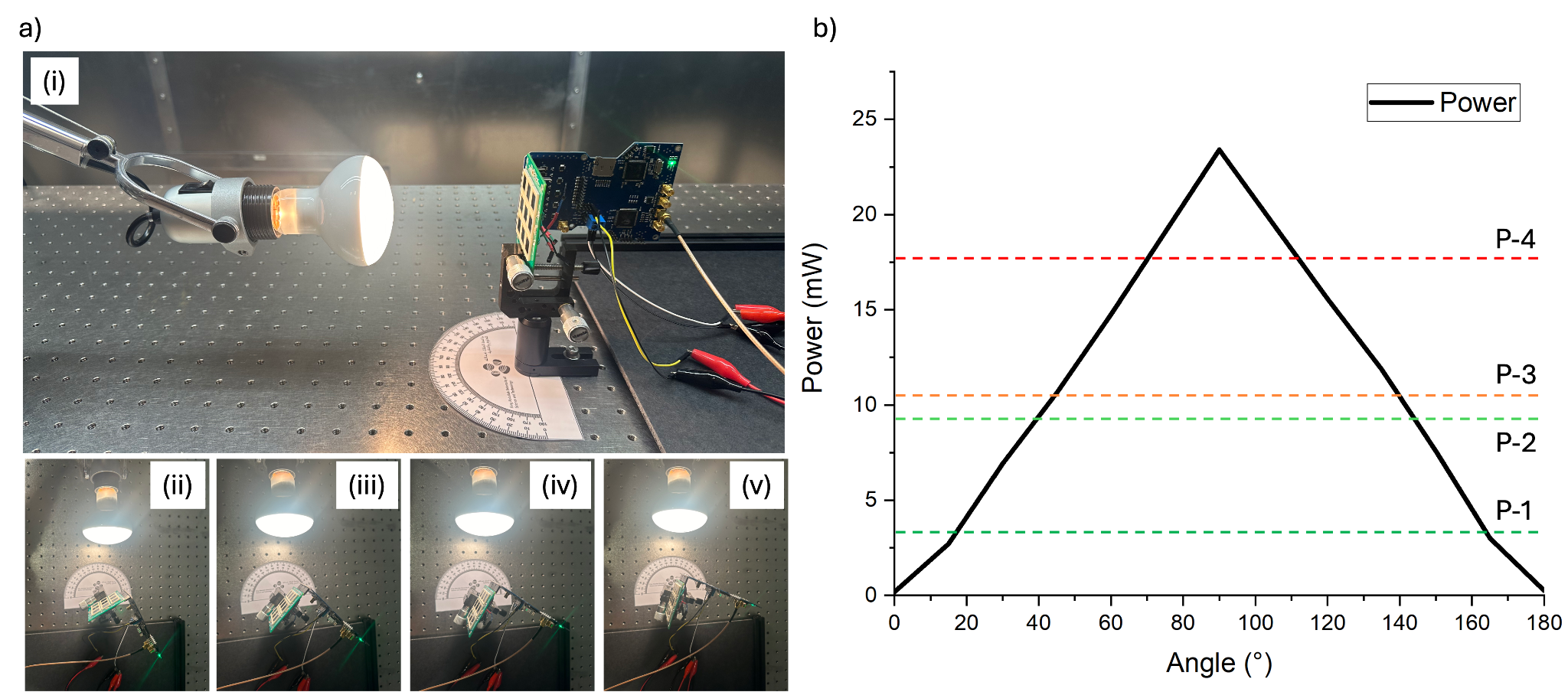


Figure S13. Self-powering batteryless measurement under different illumination angles. a) Spotlight setup with (i) direct line-of-sight and angles of (ii) 60°, (iii) 45°, (iv) 30°, and (v) 15°. b) Power generated at different angles and power modes sustained while continuous harvesting.
